# A comprehensive study on cellular RNA editing activity in response to infections with different subtypes of influenza a viruses

**DOI:** 10.1186/s12864-017-4330-1

**Published:** 2018-01-19

**Authors:** Yingying Cao, Ruiyuan Cao, Yaowei Huang, Hongxia Zhou, Yuanhua Liu, Xuan Li, Wu Zhong, Pei Hao

**Affiliations:** 1 Key Laboratory of Molecular Virology and Immunology, Institute Pasteur of Shanghai, Chinese Academy of Sciences, Shanghai, 20031 China; 2 National Engineering Research Center For the Emergence Drugs, Beijing Institute of Pharmacology and Toxicology, Beijing, 100850 China; 3 Key Laboratory of Synthetic Biology, CAS Center for Excellence in Molecular Plant Sciences, Institute of Plant Physiology and Ecology, Shanghai Institutes for Biological Sciences, Chinese Academy of Sciences, Shanghai, 20032 China

**Keywords:** Influenza a virus, RNA editing, Antiviral response, Innate immunity, ADAR, APOBEC

## Abstract

**Background:**

RNA editing is an important mechanism that expands the diversity and complexity of genetic codes. The conversions of adenosine (A) to inosine (I) and cytosine (C) to uridine (U) are two prominent types of RNA editing in animals. The roles of RNA editing events have been implicated in important biological pathways. Cellular RNA editing activity in response to influenza A virus infection has not been fully characterized in human and avian hosts. This study was designed as a big data analysis to investigate the role and response of RNA editing in epithelial cells during the course of infection with various subtypes of influenza A viruses.

**Results:**

Using a bioinformatics pipeline modified from our previous study, we characterized the profiles of A-to-I and C-to-U RNA editing events in human epithelial cells during the course of influenza A virus infection. Our results revealed a striking diversity of A-to-I RNA editing activities in human epithelial cells in responses to different subtypes of influenza A viruses. The infection of H1N1 and H3N2 significantly up-regulated normalized A-to-I RNA editing levels in human epithelial cells, whereas that of H5N1 did not change it and H7N9 infection significantly down-regulated normalized A-to-I editing level in A549 cells. Next, the expression levels of ADAR and APOBEC enzymes responsible for A-to-I and C-to-U RNA editing during the course of virus infection were examined. The increase of A-to-I RNA editing activities in infections with some influenza A viruses (H1N1 and H3N2) is linked to the up-regulation of ADAR1 but not ADAR2. Further, the pattern recognition receptors of human epithelial cells infected with H1N1, H3N2, H5N1 and H7N9 were examined. Variable responsive changes in gene expression were observed with RIG-I like receptors and Toll like receptors. Finally, the effect of influenza A virus infection on cellular RNA editing activity was also analyzed in avian hosts.

**Conclusion:**

This work represents the first comprehensive study of cellular RNA editing activity in response to different influenza A virus infections in human and avian hosts, highlighting the critical role of RNA editing in innate immune response and the pathogenicity of different subtypes of influenza A viruses.

**Electronic supplementary material:**

The online version of this article (10.1186/s12864-017-4330-1) contains supplementary material, which is available to authorized users.

## Background

Influenza A viruses are among the most common and the most significant causes of respiratory diseases in a broad range of animals from avian to human hosts because of the high morbidity and mortality of some subtypes of influenza A viruses [1–3]. Influenza A viruses can be divided into subtypes on the basis of genes coding for the major surface glycoproteins hemagglutinin (HA) and neuraminidase (NA), and are comprised of a large variety of distinct subtypes with different HA and NA combinations. Until now, there are 18 known HA and 11 known NA subtypes identified [4]. Influenza A viruses are global threats to human health. In the twentieth century, influenza A viruses of H1N1, H3N2 subtypes caused pandemics in 1918 and 1968, respectively [5–7]. The H1N1 virus of swine origin (pH1N1) spread rapidly across the world and caused the 2009 pandemic [8]. Highly pathogenic avian influenza (HPAI) viruses, especially H5N1 which can be transmitted from animals to humans have caused severe disease and death in an ever-increasing number of individuals since 1997 [9–11], of which approximately 60% had a fatal outcome [12]. However, compared to H5N1 which can cause high mortality, low pathogenic avian influenza A (LPAI) H5N2 may cause no disease or mild illness and may not be detected. Recently, another novel influenza A subtype H7N9 has spread in avian and human hosts in China since March 2013. On April 5 2017, World Health Organization has reported 1364 human infections with the H7N9 virus in China, of which approximately 40% had a fatal outcome [13]. Thus, it is critical to determine how different subtypes of influenza A viruses interact with and induce differential responses from hosts.

Influenza A virus invasion triggers a number of cellular responses and alters the host transcriptome [14–16]. RNA editing is an important mechanism that expands the diversity and complexity of genetic codes [17–19]. Various types of RNA editing have been observed from bacteria, plants to humans [20, 21]. Base substitutions by deamination of adenine (A) to inosine (I) catalyzed by the adenosine deaminases acting on RNA (ADAR) enzymes [22, 23] or cytidine to uracil (C-to-U) mediated by the apolipoprotein B mRNA editing complexes (APOBECs) are the major types of RNA editing in higher eukaryotes [24, 25]. I and U are recognized as guanosine (G) and thymine(T) by the cellular machinery, respectively, during messenger RNA translation and reverse transcription. RNA editing is highly regulated, and aberrant RNA editing can have diverse effects on various cellular pathways, including responses to viral infection and innate immunity [26]. Previous studies have shown that the host enzyme-mediated editing of viral genomes can change the base composition and structure of the viral RNA [27–30]. However, cellular RNA editing activity in response to influenza A virus infection has not been fully characterized.

During viral infection, the innate immune system of the host is meant to act as a first line defense against viral invasion. The host innate immune system has the ability to recognize viral nucleic acids as invader. For example, viral double-stranded RNA (dsRNA) or single-stranded RNA (ssRNA) with a 5′-triphosphate, which are typical products of viral replication, can be detected by the cytoplasmic retinoic acid-inducible gene I (RIG-I) like receptors (RLRs) [31–34] and Toll-like receptors (TLRs) [35] that are vital to initiate anti-viral responses. The RIG-I like receptors consist of three members, including DDX58/RIG-I (retinoic acid-inducible gene I), IFIH1/MDA-5 (melanoma differentiation-associated gene 5), and DHX58/LGP-2 (laboratory of genetics and physiology 2).They can sense viral RNA and induce powerful antiviral and pro-inflammatory gene expressions [32–34]. Among various Toll-like receptors (TLRs), TLR2 and TLR3 play a significant role in the modulation of virus-mediated innate immune response and trigger anti-viral signal pathways [36–39]. However, the mechanism and role of RNA editing changes during viral infection are yet to be completely elucidated.

The development of high-throughput sequencing technology [40, 41] and the open access data sharing in genomic research enabled unprecedented resources for transcriptome sequencing data from samples infected with different subtypes of influenza A viruses. It is made possible to implement big data analysis to investigate the details and roles of RNA editing activities in response to influenza viral infection. To examine the change of RNA editing activities in hosts infected with different subtypes of influenza A viruses, we assembled a study using collected transcriptome sequencing data from human bronchial epithelial (HBE) cells infected with H1N1 and from human tracheobronchial epithelial (HTBE) cells infected with H1N1, H3N2 and H5N1, in addition to self-generated RNA-seq data by performing experiments with A549 cells (adenocarcinomic human alveolar basal epithelial cell line) infected with H7N9. Avian being natural hosts of influenza A virus, it remained unclear how cellular RNA editing activities response to various subtypes of influenza A virus infections in avian. So we collected RNA-seq data available from ileum and lung tissues of chicken and quail infected with influenza A virus subtypes, H5N1 and H5N2. With these data we characterized the RNA editing activities of human and avian hosts infected with different subtypes of influenza A viruses and analyzed the possible link to other factors of the innate immune pathway. This work represents a comprehensive study of cellular RNA editing activity in response to various influenza A virus infections in human and avian hosts at an unprecedented scale, offering novel insights into the role of RNA editing in viral host interaction and the mechanism of pathogenicity of different subtypes of influenza A viruses.

## Results

### Global profiles of A-to-I and C-to-U RNA-editing events in human lung and tracheobronchial epithelial cells

To characterize A-to-I and C-to-U RNA editing activities in human epithelial cells upon infection of influenza A viruses, we first sought to compile and collect transcriptome sequencing data from human samples with infected influenza A virus of various types. We identified from NCBI (National Center for Biotechnology Information) database RNA-seq data from human lung and tracheobronchial epithelial cells infected with different subtypes of influenza A viruses, H1N1, H3N2, H5N1 and H7N9 (Additional file 1: Table S1). They included RNA-seq data from human bronchial epithelial (HBE) cells infected with H1N1 (PR/8/34) and from human tracheobronchial epithelial (HTBE) cells infected with H1N1, H3N2 and H5N1, respectively. We also performed experiment to infect A549 cells with H7N9 and obtained its RNA-seq data (See *Methods*).

**Fig. 1:**
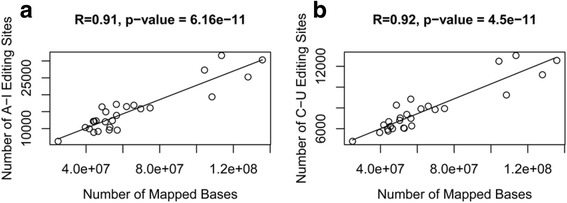
Correlation between the number of mapped RNA-seq bases and the number of RNA editing sites. **a** Correlation between the number of total mapped RNA-seq bases and the number of A-to-I RNA editing sites across all conditions. **b** Correlation between the number of total mapped RNA-seq bases and the number of C-to-U RNA editing sites across all conditions

To call A-to-I and C-to-U RNA editing events from mapped RNA-seq data, we used a modified pipeline that applied a series of filters to remove the noises (details can be seen in *Methods*), which was similar to the methods described previously [42, 43]. Comparing RNA editing activities across different conditions, we found the number of A-to-I and C-to-U RNA editing events varied among different treatments (Table 1), ranging from 4308 A-to-I editing events for HTBE cells (12 h post infection with H5N1) and 3442 C-to-U editing events for NHBE cells (at 0 h with H1N1 infection) to 39,929 A-to-I editing events for A549 cells (at 0 h with H7N9 infection) and 12,917 C-to-U editing events for HTBE cells (18 h post infection with H1N1). We found the variable number of both A-to-I and C-to-U RNA editing events has a strong linear correlation with the numbers of mapped reads across all the conditions (Fig. 1a, Pearson’s correlation *R* = 0.91, *p* = 6.16e-11, Fig. 1b, Pearson’s correlation *R* = 0.92, *p* = 4.5e-11), which was consistent with previous results [44]. These results suggested that the number of RNA editing sites showed no significant bias toward certain conditions, confirming the validity of the RNA editing events we identified throughout our pipeline.

**Table 1:** Summary of RNA-editing sites in human lung and tracheobronchial epithelial cells

Subtype	Host	Time Postinfection	Sequence Strategy	Editing Type	No. of Editing Sites	Validated Sites from Replicates
H1N1	NHBE cells	0 h rep1	Paired end	A-to-I	10,784	8735
H1N1	NHBE cells	0 h rep2	Paired end	A-to-I	11,748	
H1N1	NHBE cells	08 h rep1	Paired end	A-to-I	11,129	9619
H1N1	NHBE cells	08 h rep2	Paired end	A-to-I	18,165	
H1N1	NHBE cells	24 h rep1	Paired end	A-to-I	10,570	7506
H1N1	NHBE cells	24 h rep2	Paired end	A-to-I	14,475	
H1N1	NHBE cells	0 h rep1	Paired end	C-to-U	3442	424
H1N1	NHBE cells	0 h rep2	Paired end	C-to-U	3856	
H1N1	NHBE cells	08 h rep1	Paired end	C-to-U	4110	522
H1N1	NHBE cells	08 h rep2	Paired end	C-to-U	5626	
H1N1	NHBE cells	24 h rep1	Paired end	C-to-U	3722	453
H1N1	NHBE cells	24 h rep2	Paired end	C-to-U	4823	
H7N9	A549cells	0 h rep1	Paired end	A-to-I	35,070	8352
H7N9	A549 cells	0 h rep2	Paired end	A-to-I	39,929	
H7N9	A549 cells	1.5 h rep1	Paired end	A-to-I	29,145	5650
H7N9	A549 cells	1.5 h rep2	Paired end	A-to-I	27,806	
H7N9	A549 cells	3 h rep1	Paired end	A-to-I	25,280	6206
H7N9	A549 cells	3 h rep2	Paired end	A-to-I	30,088	
H7N9	A549 cells	7 h rep1	Paired end	A-to-I	34,678	6855
H7N9	A549 cells	7 h rep2	Paired end	A-to-I	39,780	
H7N9	A549 cells	0 h rep1	Paired end	C-to-U	5920	1437
H7N9	A549 cells	0 h rep2	Paired end	C-to-U	6329	
H7N9	A549 cells	1.5 h rep1	Paired end	C-to-U	5384	1214
H7N9	A549 cells	1.5 h rep2	Paired end	C-to-U	6796	
H7N9	A549 cells	3 h rep1	Paired end	C-to-U	5650	1209
H7N9	A549 cells	3 h rep2	Paired end	C-to-U	5531	
H7N9	A549 cells	7 h rep1	Paired end	C-to-U	7577	1572
H7N9	A549 cells	7 h rep2	Paired end	C-to-U	9198	
H1N1	HTBE cells	03 h rep1	Paired end	A-to-I	8781	5732
H1N1	HTBE cells	03 h rep2	Paired end	A-to-I	7770	
H1N1	HTBE cells	06 h rep1	Paired end	A-to-I	17,190	16,329
H1N1	HTBE cells	06 h rep2	Paired end	A-to-I	23,656	
H1N1	HTBE cells	12 h rep1	Paired end	A-to-I	21,626	17,912
H1N1	HTBE cells	12 h rep2	Paired end	A-to-I	23,818	
H1N1	HTBE cells	18 h rep1	Paired end	A-to-I	26,027	20,066
H1N1	HTBE cells	18 h rep2	Paired end	A-to-I	26,912	
H1N1	HTBE cells	03 h rep1	Paired end	C-to-U	4806	651
H1N1	HTBE cells	03 h rep2	Paired end	C-to-U	5550	
H1N1	HTBE cells	06 h rep1	Paired end	C-to-U	8484	905
H1N1	HTBE cells	06 h rep2	Paired end	C-to-U	11,521	
H1N1	HTBE cells	12 h rep1	Paired end	C-to-U	10,666	991
H1N1	HTBE cells	12 h rep2	Paired end	C-to-U	11,537	
H1N1	HTBE cells	18 h rep1	Paired end	C-to-U	12,286	1000
H1N1	HTBE cells	18 h rep2	Paired end	C-to-U	12,917	
H3N2	HTBE cells	03 h rep1	Paired end	A-to-I	18,094	13,873
H3N2	HTBE cells	03 h rep2	Paired end	A-to-I	15,234	
H3N2	HTBE cells	06 h rep1	Paired end	A-to-I	14,735	11,367
H3N2	HTBE cells	06 h rep2	Paired end	A-to-I	18,793	
H3N2	HTBE cells	12 h rep1	Paired end	A-to-I	8237	4809
H3N2	HTBE cells	12 h rep2	Paired end	A-to-I	9272	
H3N2	HTBE cells	18 h rep1	Paired end	A-to-I	10,598	5604
H3N2	HTBE cells	18 h rep2	Paired end	A-to-I	10,947	
H3N2	HTBE cells	03 h rep1	Paired end	C-to-U	8402	729
H3N2	HTBE cells	03 h rep2	Paired end	C-to-U	7545	
H3N2	HTBE cells	06 h rep1	Paired end	C-to-U	7316	715
H3N2	HTBE cells	06 h rep2	Paired end	C-to-U	9787	
H3N2	HTBE cells	12 h rep1	Paired end	C-to-U	5503	484
H3N2	HTBE cells	12 h rep2	Paired end	C-to-U	6151	
H3N2	HTBE cells	18 h rep1	Paired end	C-to-U	7855	642
H3N2	HTBE cells	18 h rep2	Paired end	C-to-U	7308	
H5N1	HTBE cells	03 h rep1	Paired end	A-to-I	8773	6196
H5N1	HTBE cells	03 h rep2	Paired end	A-to-I	9455	
H5N1	HTBE cells	06 h rep1	Paired end	A-to-I	8604	2894
H5N1	HTBE cells	06 h rep2	Paired end	A-to-I	5567	
H5N1	HTBE cells	12 h rep1	Paired end	A-to-I	6891	1287
H5N1	HTBE cells	12 h rep2	Paired end	A-to-I	4308	
H5N1	HTBE cells	18 h rep1	Paired end	A-to-I	7486	1295
H5N1	HTBE cells	18 h rep2	Paired end	A-to-I	5692	
H5N1	HTBE cells	03 h rep1	Paired end	C-to-U	5854	604
H5N1	HTBE cells	03 h rep2	Paired end	C-to-U	6061	
H5N1	HTBE cells	06 h rep1	Paired end	C-to-U	6482	381
H5N1	HTBE cells	06 h rep2	Paired end	C-to-U	4748	
H5N1	HTBE cells	12 h rep1	Paired end	C-to-U	5914	282
H5N1	HTBE cells	12 h rep2	Paired end	C-to-U	4152	
H5N1	HTBE cells	18 h rep1	Paired end	C-to-U	7014	302
H5N1	HTBE cells	18 h rep2	Paired end	C-to-U	5430

### Effects of influenza a virus infection on RNA editing patterns in human lung and tracheobronchial cells

To explore the effect of influenza A virus infection on RNA editing activities in human epithelial cells, we chose the events of common editing sites that were found in public databases and were shared by all samples along the course of virus infection (Table 1; Additional file 2: Figure S1). The RNA editing activities were measured with the normalized RNA editing level [45] of common editing sites (See *Methods*).

**Fig. 2:**
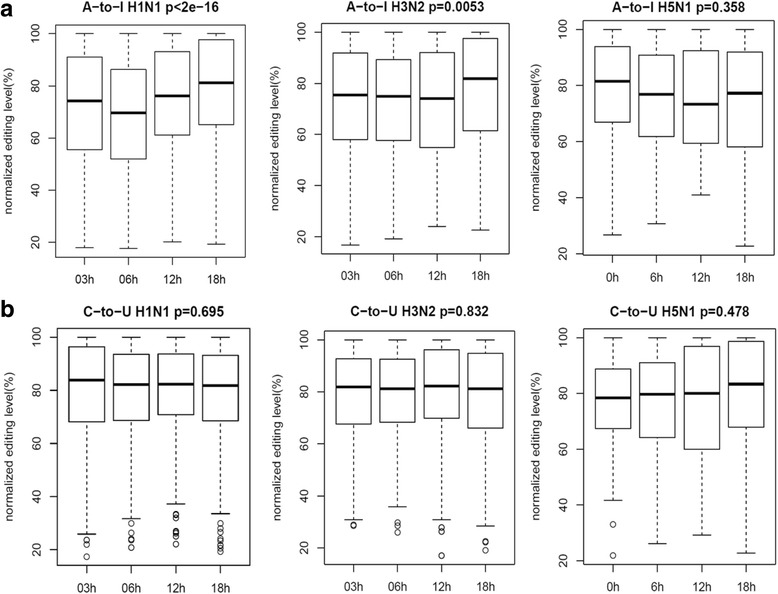
The A-to-I and C-to-U RNA editing levels of common editing sites of HTBE cells infected with H1N1, H3N2 and H5N1 at different time points. **a** The normalized A-to-I RNA editing level of HTBE cells infected with H1N1, H3N2 and H5N1 at different time points. **b** The normalized C-to-U RNA editing level of HTBE cells infected with H1N1, H3N2 and H5N1 at different time points. The normalized RNA editing level is achieved by dividing the original value of RNA editing level (which is defined as the proportion of edited reads among the total mapped reads at a given site) by the highest value at different time points

We first analyzed the RNA editing activities of HTBE cells infected with H1N1, H3N2 and H5N1 at different time points (Fig. 2). Normalized C-to-U editing levels showed no significant changes among the time points during the course of H1N1, H3N2, and H5N1 infections (Fig. 2b). One the other hand, A-to-I editing levels had significant increase along the course of infection for H1N1, H3N2, but not H5N1, in HTBE cells (Fig. 2a). For example, A-to-I editing levels for H1N1 infected cells increased significantly (*p* < 2e-16) with median level from ~70% at 3 h post-infection changing to ~80% at 24 h post-infection (Fig. 2a). The HBE cells infected with H1N1 virus confirmed what was observed in HTBE cells infected with the same subtype (Additional file 3: Figure S2). In contrast, there was no significant change in the A-to-I editing level in H5N1 infected HTBE cells (Fig. 2a).

**Fig. 3:**
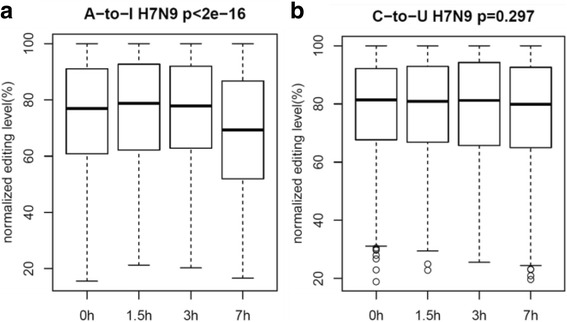
The A-to-I and C-to-U RNA editing levels of common editing sites of A549 cells infected with H7N9 at different time points. **a** The normalized A-to-I RNA editing level of A549 cells infected with H7N9 at different time points. **b** The normalized C-to-U RNA editing level of A549 cells infected with H7N9 at different time points

Using RNA-seq data we generated from H7N9 infected A549 cells, we observed a significant decrease of normalized A-to-I editing levels at common sites (Fig. 3a), forming a striking contrast with those observed in HTBE cells infected with H1N1, H3N2 and H5N1. But C-to-U RNA editing levels remained stable (Fig. 3b), which was similar to what was observed in HTBE cells infected with H1N1, H3N2 and H5N1 (Fig. 2b).

### Expression profiles of ADAR and APOBEC enzymes in human lung and tracheobronchial epithelial cells infected with influenza a viruses

**Fig. 4:**
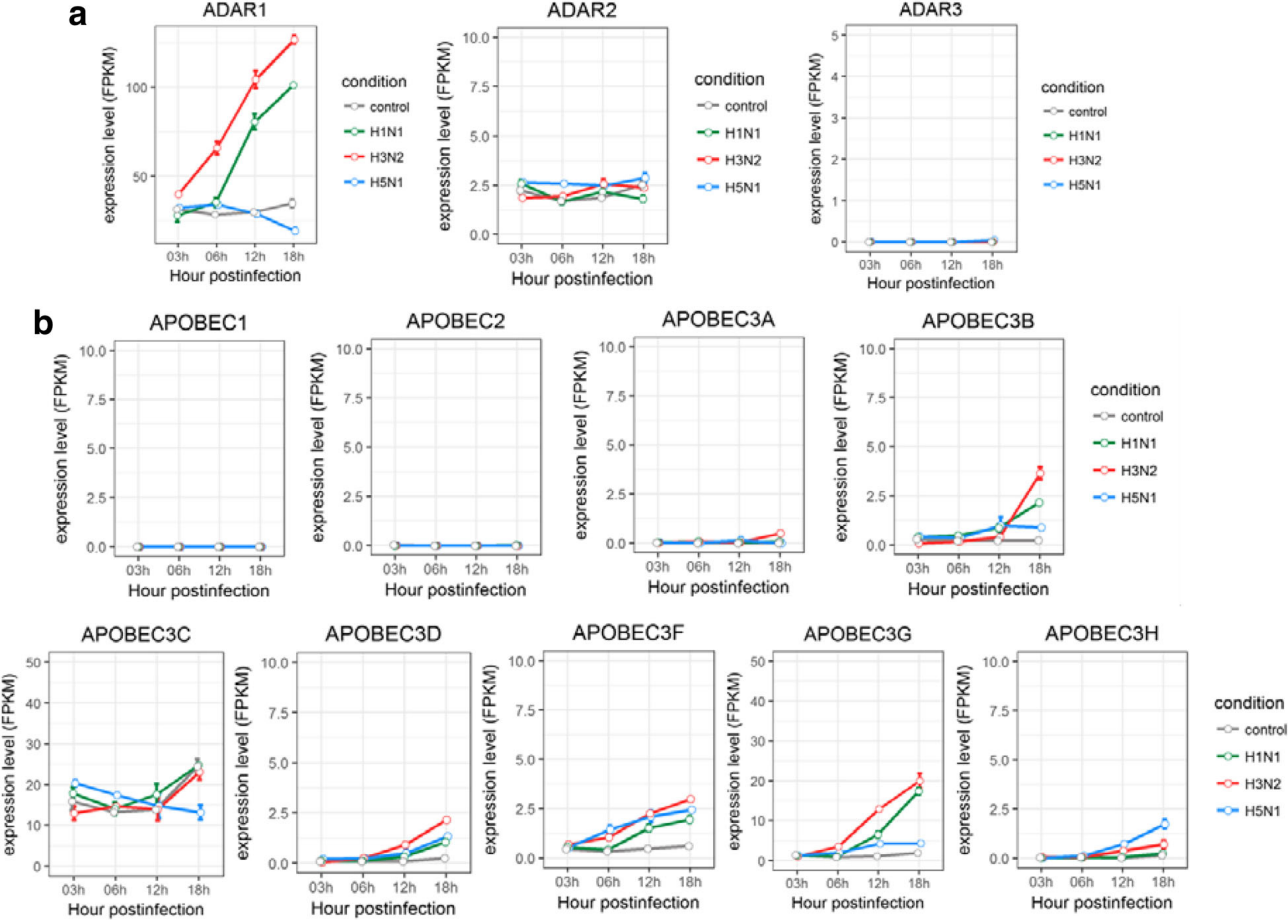
Expression profiles of ADAR and APOBEC enzymes in HTBE cells infected with H1N1, H3N2 and H5N1. **a** The expression levels of ADARs in H1N1, H3N2 and H5N1 infected HTBE cells, and uninfected HTBE cells at different time points. **b** The expression levels of APOBECs in H1N1, H3N2 and H5N1 infected HTBE cells, and uninfected HTBE cells at different time points. The gene expression is measured using FPKM reported by Cufflinks (Version: 2.2.1)

To identify the molecular determinants underlying the different RNA editing activities under influenza A virus infection, we performed differential gene expression analysis for ADAR and APOPEC enzymes, which were responsible for A-to-I and C-to-U RNA editing during the course of virus infections, in human lung and tracheobronchial epithelial cells. Cufflinks software (Version: 2.2.1) was used to quantify the gene expression level measured by FPKM (fragments per kilobase of transcript per million mapped reads) and to identify the differentially expressed genes between control and virus-infected groups. For expression of ADAR and APOBEC genes in HTBE cells infected with H1N1, H3N2, or H5N1 (Fig. 4), we found that ADAR1 expression increased dramatically along the course of infection for H1N1 and H3N2, but not H5N1, which was consistent with the changes of A-to-I RNA editing levels in H1N1, H3N2, and H5N1 infected cells. On the other hand, there were no significant changes in ADAR2 or ADAR3 expression under all condition, suggesting ADAR2 and ADAR3 genes were not responsive to influenza virus A infections.

Differential expressions were observed for APOBEC3 family of enzymes during the course of influenza virus A infections, but not for APOBEC1 or APOBEC2 (Fig. 4b). Similar to ADAR1 gene, APOBEC3 family displayed differential response to the infection of H1N1, H3N2, or H5N1. For example, the expression levels of APOBEC3B and AOPBEC3G were much higher in H1N1 or H3N2 infected cells than in H5N1 infected cells. Since no significant change was observed in C-to-U editing levels in HTBE cells infected with H1N1, H3N2, or H5N1, these intriguing results indicated that APOBEC3s were responded to influenza virus infection, but had no observed influence on the C-to-U editing of HTBE cells. The results of ADAR and APOBEC genes from HTBE cells infected with H1N1 were similar to those of HBE cells infected with the same influenza virus, H1N1 (Additional file 4: Figure S3).

**Fig. 5:**
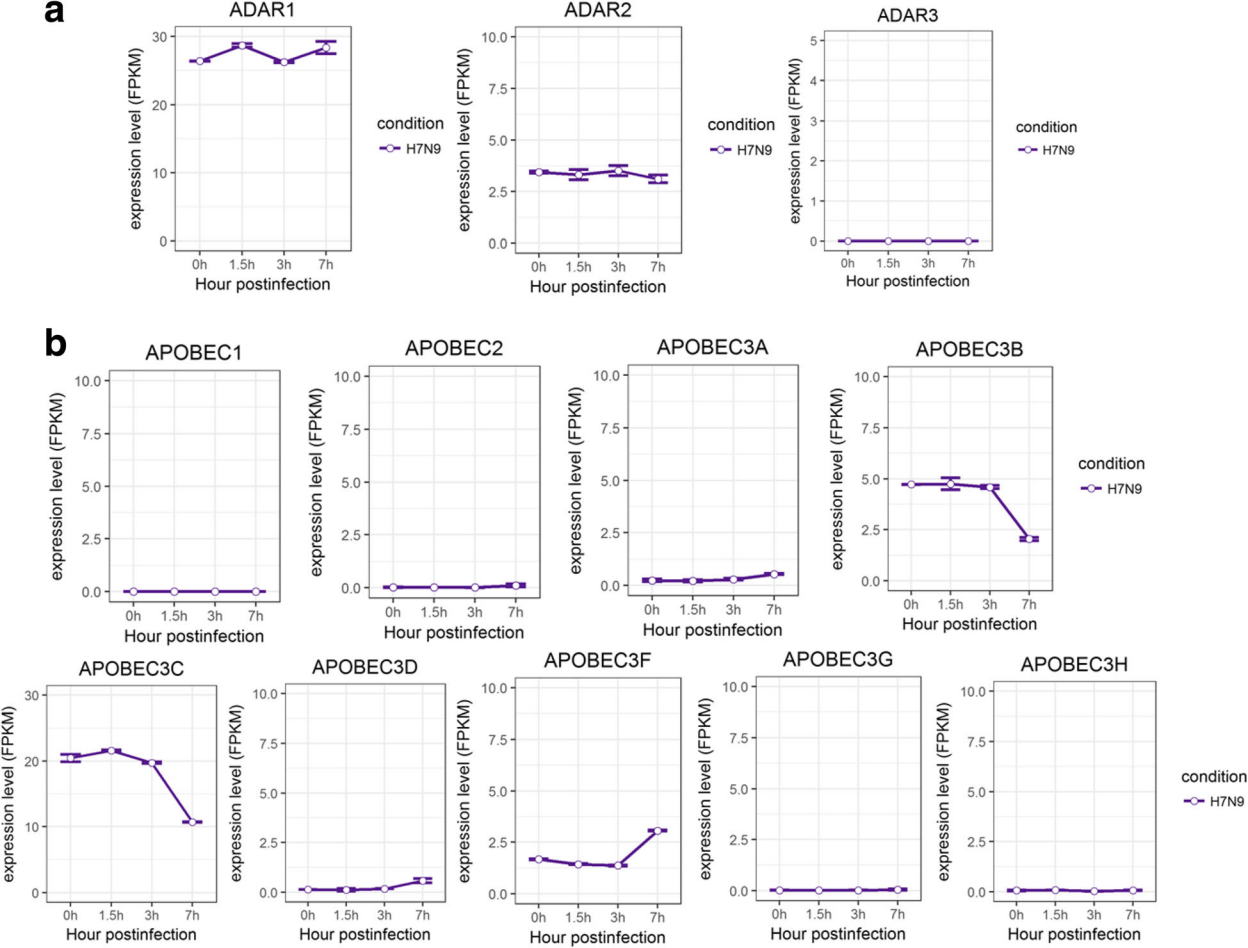
Expression profiles of ADAR and APOBEC enzymes in A549 cells infected with H7N9. **a** The expression levels of ADARs in H7N9 infected A549 cells at different time points. **b**) The expression levels of APOBECs in H7N9 infected A549 cells at different time points. The gene expression is measured using FPKM reported by Cufflinks (Version: 2.2.1)

Then we analyzed the expression ADAR and APOBEC enzymes using RNA-seq data we generated from H7N9 infected A549 cells. No significant change was observed with the expression of ADAR1–3, APOBEC1, APOBEC2, and APOBEC3A 3D, 3G, and 3H, whereas APOBEC3B, and 3C had a significant decrease, and APOBEC3F had a significant increase in A549 cells during the course of infection with H7N9 (Fig. 5). The unchanged levels of ADARs were roughly consistent with the activities of A-to-I editing along the course of H7N9 infection, which showed a slight decrease (Fig. 3a). Again the change (here decrease) in expression of APOBEC3 family of genes did not induce visible changes in C-to-U editing activities (Fig. 3b) under H7N9 infections, similar to what was observed in C-to-U editing levels in HTBE cells infected with H1N1, H3N2, or H5N1.

### Expression profiles of pattern recognition receptors in human lung and tracheobronchial epithelial cells infected with influenza a viruses

**Fig. 6:**
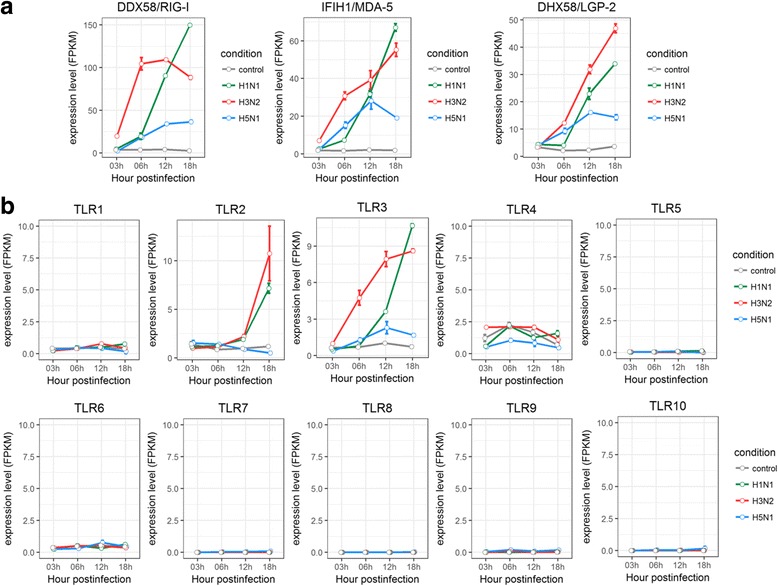
Expression profiles of Pattern Recognition Receptors in HTBE cells infected with H1N1, H3N2 and H5N1. **a** The expression levels of RLRs in H1N1, H3N2 and H5N1 infected HTBE cells, and uninfected HTBE cells at different time points. **b** The expression levels of TLRs in H1N1, H3N2 and H5N1 infected HTBE cells, and uninfected HTBE cells at different time points. The gene expression is measured using FPKM reported by Cufflinks (Version: 2.2.1)

**Fig. 7:**
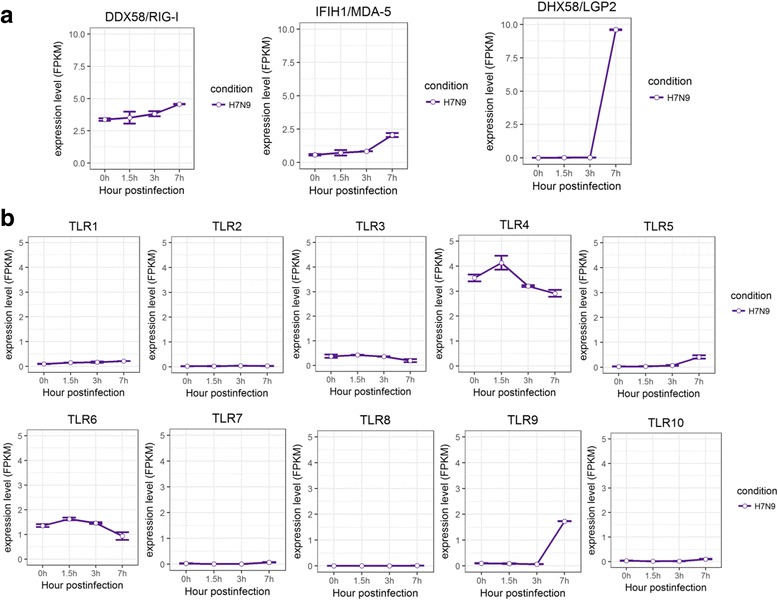
Expression profiles of Pattern Recognition Receptors in A549 cells infected with H7N9. **a** The expression levels of RLRs in H7N9 infected A549 cells at different time points. **b** The expression levels of TLRs in H7N9 infected A549 cells at different time points. The gene expression is measured using FPKM reported by Cufflinks (Version: 2.2.1)

Since detection of viral RNAs by Pattern Recognition Receptors (PRRs) is the first step for initiating anti-viral responses, we further examined the expression changes of of PRR genes triggered by different subtypes of influenza A viruses in human lung and tracheobronchial epithelial cells. In comparison with control group, there was a general increase in the gene expression of RIG-I like receptors, including DDX58/RIG-I, IFIH1/MDA-5, DHX58/LGP-2 in human lung and tracheobronchial epithelial cells infected with H1N1, H3N2, H5N1, or H7N9 (Figs. 6a, 7a, and Additional file 4: Figure S4). However, different expression patterns of Toll like receptors were observed for cells infected with H1N1, H3N2, H5N1, or H7N9. The expression of TLR2 and TLR3 were up-regulated in H1N1 and H3N2 infected HBE and HTBE cells, whereas H5N1 induced slight uptick in TLR3 expression and H7N9 induced increased expression for TLR9 but downward trend for TLR4 and TLR6 (Figs. 6b, 7b, and Additional file 4: Figure S4). Taken together, different subtypes of influenza A viruses induced distinct expression patterns of PRR genes.

### Effects of influenza a virus infections on RNA editing activity in ileum and lung tissues of chicken and quail

**Fig. 8:**
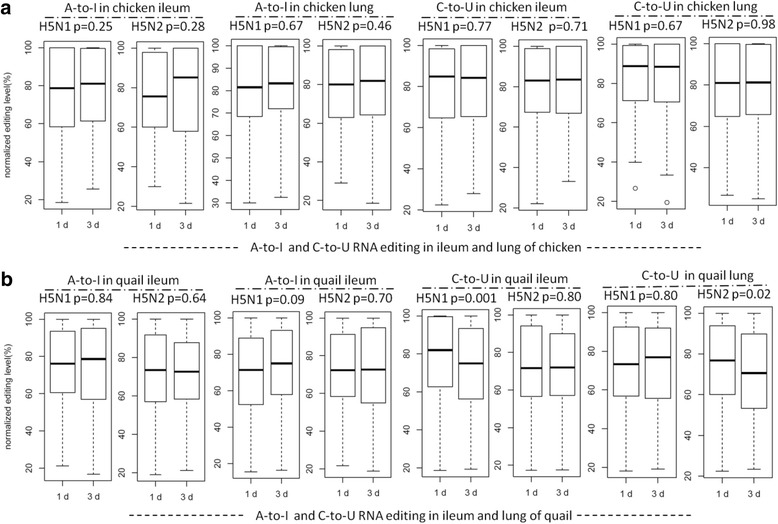
The A-to-I and C-to-U RNA editing levels of common editing sites of ileum and lung of chicken and quail infected with H5N1 and H5N2 at different time points. **a** The normalized A-to-I and C-to-U RNA editing level of ileum and lung of chicken infected with H5N1 and H5N2 at different time points. (**b**) The normalized A-to-I and C-to-U RNA editing level of ileum and lung of quail infected with H5N1 and H5N2 at different time points. The normalized RNA editing level is achieved by dividing the original value of RNA editing level (which is defined as the proportion of edited reads among the total mapped reads at a given site) by the highest value at different time points.

Avian are natural hosts of influenza A virus. It remained undetermined how cellular RNA editing activities response to various subtypes of influenza A virus infections in avian hosts. Upon search on NCBI databases, we found transcriptome sequencing data from ileum and lung tissues of chicken and quail infected with influenza A virus subtypes, H5N1 and H5N2 (Table 1). Their A-to-I and C-to-U editing activities were analyzed along the course of influenza virus infections. No significant changes in A-to-I or C-to-U RNA editing activity were observed in ileum and lung tissues of either chicken or quail infected with influenza virus subtype H5N1 (with the exception of dropped C-to-U editing in quail ileum) (Fig. 8a), which was highly similar to what was found in human H5N1 infection results. For avian hosts infected with subtype H5N2, the results were found to be highly similar to those infected with subtype H5N1, with the exception of decreased C-to-U editing in quail lung (Fig. 8b).

We further analyzed the differential expression of ADAR and APOBEC enzymes in quail and chicken as described in human cells. No significant change in expression of ADAR or APOBEC enzymes was observed between controls and the virus-infected groups (Additional file 5: Table S2 and Table S3). These results were also highly consistent to those of human cells infected with H5N1. So different from the two subtypes of influenza viruses: H1N1 and H3N2, infection of H5N1 consistently induce no or very minor response in A-to-I RNA editing, expression of ADARs/APOBECs, and TLRs in both human and avian hosts.

## Discussion

RNA editing has long been thought to have diverse effects on various RNA-mediated cellular pathways, including cellar innate immune response [26]. Our study was performed using a large collection of RNA-seq data from different hosts (human, chicken, quail) infected with different subtypes of influenza A viruses (H1N1, H3N2, H5N1, H5N2, H7N9) at different time points. The differences in A-to-I and C-to-U RNA editing activities were revealed in human cells infected with different subtypes at different time points. First, the A-to-I RNA editing level increased along the course of infection with H1N1, and H3N2, and decreased with H7N9 infection. There was no significant change in A-to-I RNA editing level in H5N1 infected human cells. Second, the expression levels of ADAR1, RIG-I like receptors, TLR2 and TLR3 in H1N1 and H3N2 infected human cells are much higher than H5N1 and H7N9 infected human cells, which agrees with the patterns of A-to-I RNA editing in the according cells. These results imply that the A-to-I RNA editing plays a critical role in the process of virus infection. The responses of the host can be modulated by the rapid changes in A-to-I editing during viral infection and have a profound implication for the pathogenicity or the virulence of different subtypes of influenza A viruses. To our knowledge, it is the first time in depth to describe the different RNA editing patterns in human cells infected with different influenza A viruses as well as the different RNA editing patterns of quail and chicken infected with H5N1 and H5N2. Our analysis of the difference in RNA editing profiles of human and avian hosts infected with different subtypes of influenza A viruses adds new details to the virus-host interactions.

Recently, there was a controversy for the roles of ADAR enzymes during viral infection, and different lines of evidences indicated ADAR enzymes can play both antiviral role and also act as pro-viral proteins [46]. Curiously, ADAR1 has emerged as a replication enhancer of many viruses (HIV-1, MV, VSV, HDV) during acute viral infections [47]. However, our study showed that the failure to up-regulate ADAR1 in H5N1 infected human bronchial epithelial cells led to more viral gene transcripts of H5N1. However, with higher expression levels of ADAR1 in H1N1 and H3N2 infected human bronchial epithelial cells, fewer viral gene transcripts were observed. Hence, our results suggest that the ADAR1 has an antiviral role in human cells depending on the subtypes of influenza virus. This finding agrees with Ward et al. who demonstrated the antiviral role of ADAR1 by infecting ADAR1 p150−/− MEF cells with the influenza A WSN strain and observing that p150 isoform of ADAR1 is a restriction factor in the replication of influenza A virus [48]. Furthermore, our results also demonstrated its antiviral role depended on the subtypes of influenza A virus. However, the mechanism of this function remains to be elucidated.

Importantly, we have shown that the expression levels of PRRs are much lower in H5N1 and H7N9 infected cells, which may suggest that the failure to elicit strong early innate immune responses is crucial for the pathogenicity of H5N1 and H7N9. This result agrees with the results of previous studies [49, 50] that reported the higher virulence of H5N1 corresponded to the weaker induction of innate immune responses. Until now little was known about ADAR and APOBEC enzymes and innate immune system in avian, like chicken and quail. Even worse, many innate immunity related genes have not been identified in avian species. Our study represents first such research conducted in avian system. While it open a new gateway toward understand of the molecular determinants underlying the RNA editing activities in avian like quail and chicken, more work remains to be pursue to further our understanding of RNA editing functions in virus-avian interactions.

## Conclusion

This work represents the first comprehensive study of cellular RNA editing activity in response to different influenza A virus infections in human hosts (HBE, HTBE, and A549 cells), and in avian hosts (ileum and lung of chicken and quail), highlighting the important roles of RNA editing in innate immune response. A striking difference of cellular RNA editing activities in response to various influenza A virus subtypes were observed. The increase of A-to-I RNA editing activities in infections with some influenza A virus (H1N1 and H3N2) is linked to the up-regulation of ADAR1 but not ADAR2, through RIG-I like receptors (DDX58/RIG-I, IFIH1/MDA-5, DHX58/LGP-2) and Toll-like receptors (TLR2, TLR3) in innate immune system. Our study gives a strong functional implication of A-to-I RNA editing on the pathogenicity of different subtypes of influenza A viruses.

## Methods

### Collection of transcriptome sequencing data

The transcriptome sequencing data for the A549 cells infected with H7N9 were obtained from our laboratory. A549 cells were infected with H7N9 (A/Anhui/1/2013) at MOI of 1 for 1.5 h, 3 h and 7 h. Total RNA was isolated from the infected cells using the RNeasy Mini Kit (Qiagen; Valencia, CA). The sequencing library was prepared using Illumina TruSeq RNA sample preparation kit v2. The mRNA was derived from the total RNA using poly-T oligo-attached magnetic beads. Then the mRNA was fragmented and converted into cDNA. The adapters were ligated to the cDNA and then the fragments were amplified by PCR. Paired-end sequencing (101 × 2) was performed using Illumina Hiseq 2000. Each condition of the samples was sequenced in duplicate. The RNA-seq data have been deposited in NCBI’s Gene Expression Omnibus under accession code GSE97949.

The public transcriptome sequencing data used in this study were mainly from NCBI’s GEO (Gene Expression Omnibus) and SRA (Sequence Read Archive). The RNA sequencing data for HBE cells infected with H1N1 (PR/8/34) at MOI of 1 for 8 or 24 h were obtained from NCBI’s SRA under accession number SRP066992 (Additional file 1: Table S1). The transcriptome sequencing data for the peripheral blood samples from patients infected with H7N9 were obtained from NCBI’s SRA under accession number SRP033696 (Additional file 1: Table S1). The transcriptome sequencing data for HTBE cells infected with H1N1, H3N2 and H5N1 at MOI of 5 for 3, 6, 12, and 18 h post infection were obtained from NCBI’s SRA under accession number SRP091886 (Additional file 1: Table S1). The tissue transcriptome sequencing data for ileum and lung of chicken and quail infected with H5N1 at 101.5 EID50 and H5N2 at 106 EID50 were obtained from NCBI’s SRA. The accession number were ERP006915 and ERP009538, respectively (Additional file 1: Table S1). The reference genome and the gene annotation data for quail are downloaded from “Japanese Quail (*Coturnix japonica*) Genome Sequencing Project” website (http://viewer.shigen.info/uzura/download.php), the reference genome and the gene annotation data for human (hg19) were downloaded from Illumina’s iGenomes project (https://ccb.jhu.edu/software/tophat/igenomes.shtml). The reference genome data for different subtypes of influenza A viruses are downloaded from GISAID (Global Initiative on Sharing All Influenza Data) (http://platform.gisaid.org/epi3/).

### Sequence alignment and the identification of RNA editing sites

The workflow of this study was shown in Additional file 6: Table S4. On the first stage as shown in Additional file 6: Table S4, the RNA sequencing reads were first processed to remove adaptors using Trim_galore [51], and then the low quality reads (quality scores under 20) were removed from both 5′ and 3′ ends using Trimmomatic [52]. Then we evaluated the clean datasets with FastQC [53]. The pipeline we used for the identification of RNA editing sites (A-to-I and C-to-U) was modified from what was described previously [42]. On the second stage as shown in Additional file 6: Table S4, clean RNA sequencing reads from different species were aligned to their reference genomes using the TopHat program (TopHat v2.0.11) [54]. On the third stage as shown in Additional file 6: Table S4, we used Samtools (Version: 0.1.19) [55] mpileup program to call the RNA variants. The parameters we used were ‘-Q 20’ and default values. Then we used the following filtering criterias to filter the RNA variants and identify RNA editing sites: 1) the coverage depth of variant sites should be above2; 2) the variant frequency should be between 0.1 and 90%; 3) filter all known SNPs; 4) keep RNA variants validated by duplicates at the same time point; 5) keep RNA variants validated by the published RNA editing sites.

### Profiling gene expression levels, viral transcript counts and RNA editing levels

The aligned files we obtained with the method described above were processed with Cufflinks (Version: 2.2.1) [56] using parameters “-g genes.gtf” to get the values of gene expression levels for different hosts. FPKM values were used for the measurement of the gene expression levels. The viral transcript counts were achieved with Samtools idxstats program. The editing levels for A-to-I and C-to-U editing sites were achieved using the Samtools mpileup program. With these files we quantified the RNA editing level defined as the proportion of edited reads among the total mapped reads at a given site for each editing site of the common editing sites occurred at different time points across all conditions. The common editing sites were defined as the editing sites found in public databases and were shared by all samples along the course of infections by one subtype virus in our study (Additional file 2: Figure S1 Information of common editing sites). The RNA editing levels we calculated are then normalized by dividing the original value of RNA editing level by the highest value at different time points [45].

### Statistical analysis

Statistical analysis was performed using the computing environment R (Version: 3.3.2). Data were expressed as mean ± SE. The one way ANOVA test was used to compare the RNA editing levels of the common editing sites at different time points. Pearson correlation test was used to measure the strength of a linear association between two variables. *P* values lower than 0.05 were considered to indicate a statistically significant difference or correlation.

## Additional files

Additional file 1: Table S1. The sources of deep-sequencing transcriptome data of hosts infected with different subtypes of influenza viruses. (XLSX 14 kb)

Additional file 2: Figure S1. Information of common editing sites. (XLSX 42 kb)

Additional file 3: Figure S2. The A-to-I and C-to-U RNA editing levels of common editing sites of HBE cells infected with H1N1. (DOCX 72 kb)

Additional file 4: Figure S3. Expression profiles of ADAR and APOBEC enzymes in HBE cells infected with H1N1. **Figure S4.** Expression profiles of Pattern Recognition Receptors in HBE cells infected with H1N1. (DOCX 631 kb)

Additional file 5: Table S2. Expression profiles of ADAR and APOBEC enzymes in chicken infected with H5N1 and H5N2. **Table S3.** Expression profiles of ADAR and APOBEC enzymes in quail infected with H5N1 and H5N2. (DOCX 23 kb)

Additional file 6: Table S4. Work flow of this study. (DOCX 76 kb)
