# Supplementary material for: A comprehensive study on cellular RNA editing activity in response to infections with different subtypes of influenza a viruses
Source: BMC Genomics. 2018 Jan 19;19(Suppl 1):925. doi: 10.1186/s12864-017-4330-1 (PMC5780764; doi:10.1186/s12864-017-4330-1)

**Figure S2.** The A-to-I and C-to-U RNA editing levels of common editing sites of HBE cells infected with H1N1.


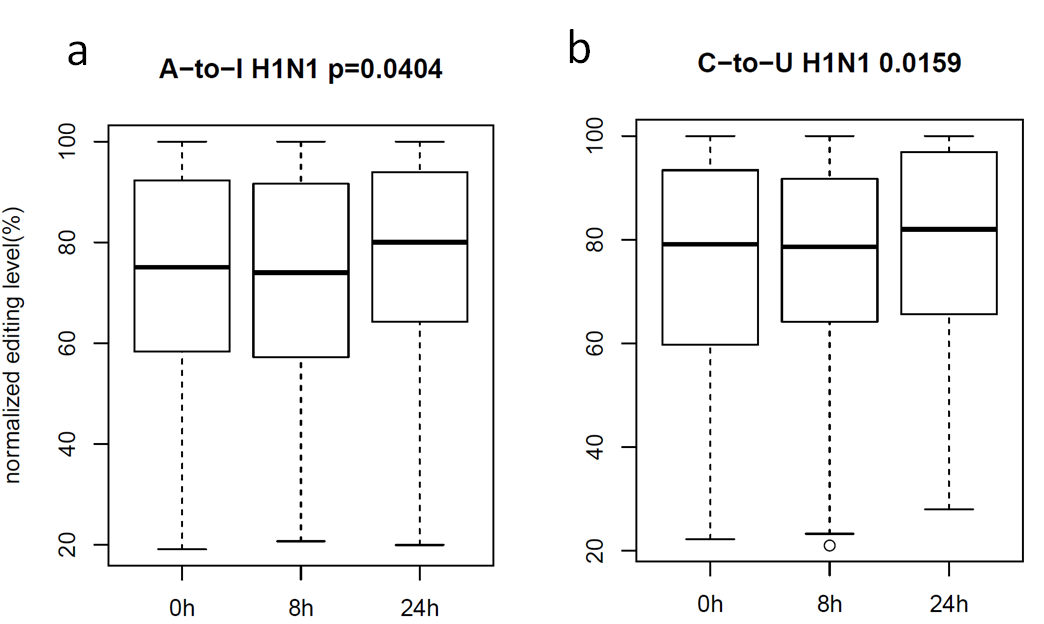

Supplement: Supplementary file 3 — The A-to-I and C-to-U RNA editing levels of common editing sites of HBE cells infected with H1N1. (DOCX 72 kb) [file 12864_2017_4330_MOESM3_ESM.docx]
