# Supplementary material for: A comprehensive study on cellular RNA editing activity in response to infections with different subtypes of influenza a viruses
Source: BMC Genomics. 2018 Jan 19;19(Suppl 1):925. doi: 10.1186/s12864-017-4330-1 (PMC5780764; doi:10.1186/s12864-017-4330-1)
Supplement: Supplementary file 4 — Expression profiles of ADAR and APOBEC enzymes in HBE cells infected with H1N1. Figure S4. Expression profiles of Pattern Recognition Receptors in HBE cells infected with H1N1. (DOCX 631 kb) [file 12864_2017_4330_MOESM4_ESM.docx]

**Figure S3.** Expression profiles of ADAR and APOBEC enzymes in HBE cells infected with H1N1.

**
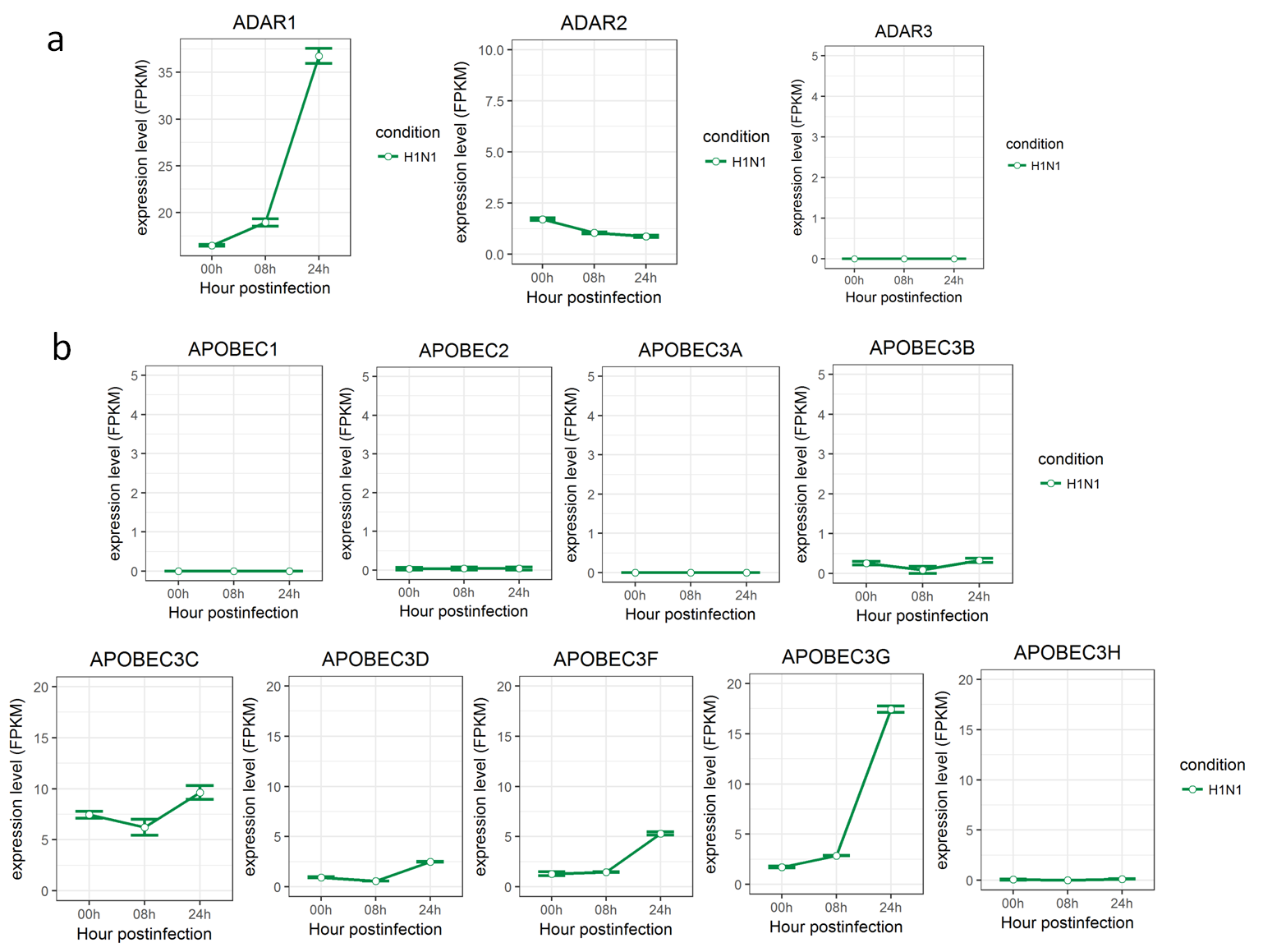
**

**Figure S4.** Expression profiles of Pattern Recognition Receptors in HBE cells infected with H1N1.

**
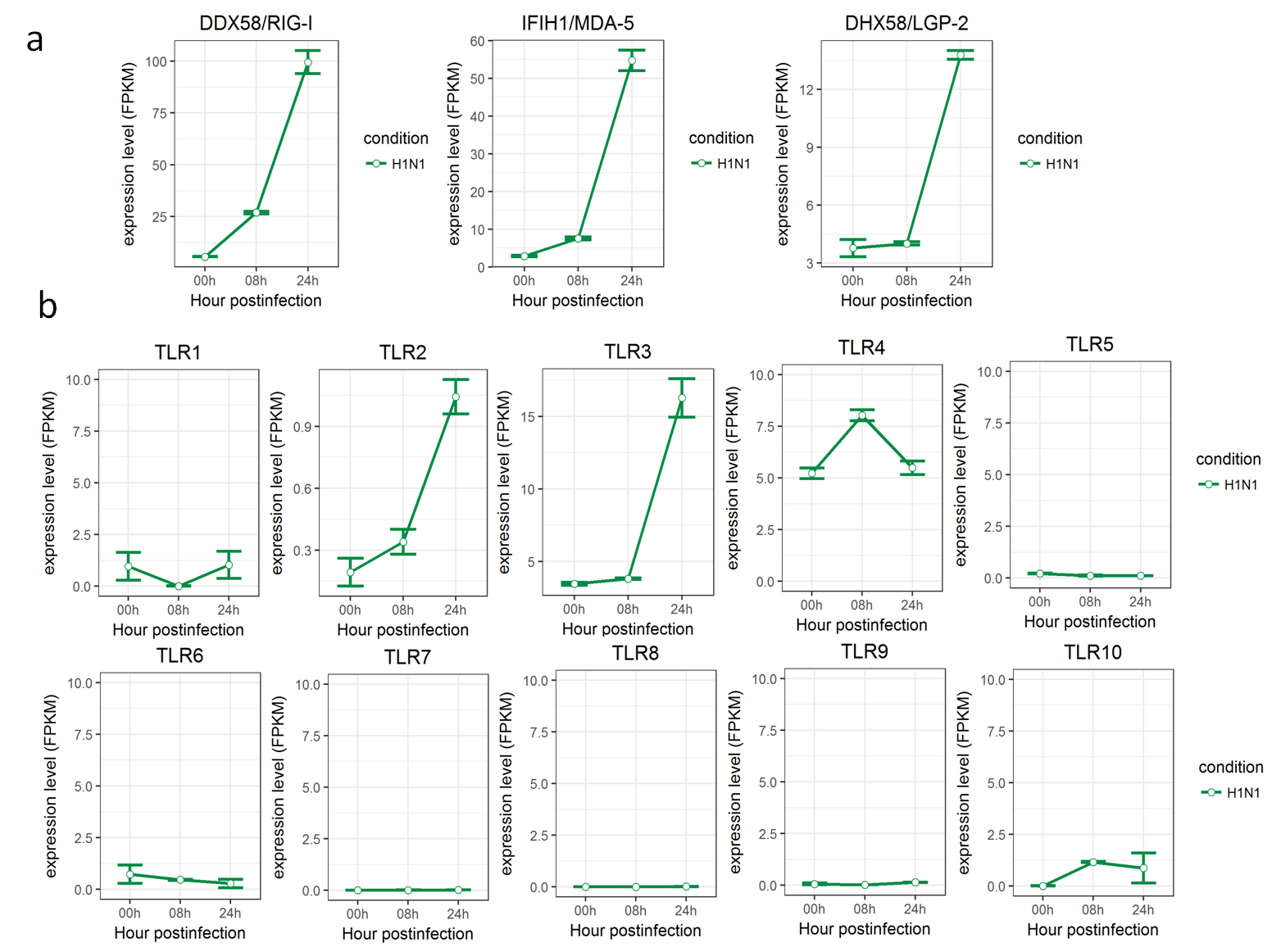
**
