# Supplementary material for: A comprehensive study on cellular RNA editing activity in response to infections with different subtypes of influenza a viruses
Source: BMC Genomics. 2018 Jan 19;19(Suppl 1):925. doi: 10.1186/s12864-017-4330-1 (PMC5780764; doi:10.1186/s12864-017-4330-1)
Supplement: Supplementary file 5 — Expression profiles of ADAR and APOBEC enzymes in chicken infected with H5N1 and H5N2. Table S3. Expression profiles of ADAR and APOBEC enzymes in quail infected with H5N1 and H5N2. (DOCX 23 kb) [file 12864_2017_4330_MOESM5_ESM.docx]

**Table S2.** Expression profiles of ADAR and APOBEC enzymes in chicken infected with H5N1 and H5N2.

| conditions | gene | sample_1 | sample_2 | status | value_1 | value_2 | log2(fold_change) | test_stat | p_value | q_value | significant |
| --- | --- | --- | --- | --- | --- | --- | --- | --- | --- | --- | --- |
| H5N1 ileum 1d | ADARB2 | none_ileum_1d | H5N1_ileum_1d | NOTEST | 0.039211 | 0.051822 | 0.402301 | 0 | 1 | 1 | no |
|  | ADARB1 | none_ileum_1d | H5N1_ileum_1d | OK | 15.8164 | 15.7963 | -0.00182959 | -0.0038 | 0.99485 | 0.998115 | no |
|  | ADAR | none_ileum_1d | H5N1_ileum_1d | OK | 21.3853 | 19.8624 | -0.106579 | -0.22816 | 0.7176 | 0.904059 | no |
|  | APOBEC4 | none_ileum_1d | H5N1_ileum_1d | NOTEST | 0.547604 | 0.687231 | 0.327663 | 0 | 1 | 1 | no |
|  | APOBEC2 | none_ileum_1d | H5N1_ileum_1d | NOTEST | 0.225923 | 0.521708 | 1.20741 | 0 | 1 | 1 | no |
| H5N1 ileum 3d | ADARB2 | none_ileum_3d | H5N1_ileum_3d | NOTEST | 0.011122 | 0.033552 | 1.59301 | 0 | 1 | 1 | no |
|  | ADARB1 | none_ileum_3d | H5N1_ileum_3d | OK | 17.3968 | 20.4508 | 0.233337 | 0.621446 | 0.28295 | 0.826022 | no |
|  | ADAR | none_ileum_3d | H5N1_ileum_3d | OK | 22.1024 | 25.9493 | 0.231496 | 0.647969 | 0.26035 | 0.804262 | no |
|  | APOBEC4 | none_ileum_3d | H5N1_ileum_3d | NOTEST | 0.598335 | 0.656671 | 0.134218 | 0 | 1 | 1 | no |
|  | APOBEC2 | none_ileum_3d | H5N1_ileum_3d | NOTEST | 0.503731 | 0.634038 | 0.331914 | 0 | 1 | 1 | no |
| H5N1 lung 1d | ADARB2 | none_lung_1d | H5N1_lung_1d | NOTEST | 0.025082 | 0.011225 | -1.15993 | 0 | 1 | 1 | no |
|  | ADARB1 | none_lung_1d | H5N1_lung_1d | OK | 6.79729 | 6.32261 | -0.104439 | -0.21386 | 0.6606 | 0.838364 | no |
|  | ADAR | none_lung_1d | H5N1_lung_1d | OK | 20.3316 | 18.9125 | -0.104378 | -0.24479 | 0.62645 | 0.818577 | no |
|  | APOBEC4 | none_lung_1d | H5N1_lung_1d | OK | 2.17623 | 1.72221 | -0.337573 | -0.37973 | 0.43585 | 0.677698 | no |
|  | APOBEC2 | none_lung_1d | H5N1_lung_1d | OK | 53.8353 | 3.21545 | -4.06546 | -2.86438 | 0.00815 | 0.061574 | no |
| H5N1 lung 3d | ADARB2 | none_lung_3d | H5N1_lung_3d | NOTEST | 0.062246 | 0.012049 | -2.36909 | 0 | 1 | 1 | no |
|  | ADARB1 | none_lung_3d | H5N1_lung_3d | OK | 7.00743 | 4.86833 | -0.525458 | -0.70361 | 0.2313 | 0.961343 | no |
|  | ADAR | none_lung_3d | H5N1_lung_3d | OK | 24.8835 | 18.5797 | -0.421465 | -0.67671 | 0.247 | 0.973746 | no |
|  | APOBEC4 | none_lung_3d | H5N1_lung_3d | OK | 1.94417 | 2.41831 | 0.314848 | 0.279005 | 0.62775 | 0.999779 | no |
|  | APOBEC2 | none_lung_3d | H5N1_lung_3d | OK | 18.4131 | 3.97658 | -2.21113 | -1.27475 | 0.2242 | 0.951535 | no |
| H5N2 ileum 1d | ADARB2 | none_ileum_1d | H5N2_ileum_1d | NOTEST | 0.040823 | 0.078773 | 0.94833 | 0 | 1 | 1 | no |
|  | ADARB1 | none_ileum_1d | H5N2_ileum_1d | OK | 16.5941 | 15.2545 | -0.121436 | -0.25848 | 0.64775 | 0.998655 | no |
|  | ADAR | none_ileum_1d | H5N2_ileum_1d | OK | 22.4468 | 31.1494 | 0.47269 | 1.01311 | 0.0837 | 0.617656 | no |
|  | APOBEC4 | none_ileum_1d | H5N2_ileum_1d | NOTEST | 0.577355 | 0.401181 | -0.525206 | 0 | 1 | 1 | no |
|  | APOBEC2 | none_ileum_1d | H5N2_ileum_1d | NOTEST | 0.236466 | 0.656347 | 1.47283 | 0 | 1 | 1 | no |
| H5N2 ileum 3d | ADARB2 | none_ileum_3d | H5N2_ileum_3d | NOTEST | 0.010754 | 0.013815 | 0.361395 | 0 | 1 | 1 | no |
|  | ADARB1 | none_ileum_3d | H5N2_ileum_3d | OK | 16.8763 | 14.556 | -0.213389 | -0.48831 | 0.36465 | 0.999395 | no |
|  | ADAR | none_ileum_3d | H5N2_ileum_3d | OK | 21.442 | 29.1297 | 0.442051 | 1.03945 | 0.05575 | 0.549057 | no |
|  | APOBEC4 | none_ileum_3d | H5N2_ileum_3d | NOTEST | 0.580719 | 0.871818 | 0.586188 | 0 | 1 | 1 | no |
|  | APOBEC2 | none_ileum_3d | H5N2_ileum_3d | NOTEST | 0.488351 | 0.541699 | 0.149572 | 0 | 1 | 1 | no |
| H5N2 lung 1d | ADARB2 | none_lung_1d | H5N2_lung_1d | NOTEST | 0.024408 | 0.018447 | -0.403985 | 0 | 1 | 1 | no |
|  | ADARB1 | none_lung_1d | H5N2_lung_1d | OK | 6.61518 | 6.8278 | 0.0456406 | 0.081699 | 0.87315 | 0.951822 | no |
|  | ADAR | none_lung_1d | H5N2_lung_1d | OK | 19.8419 | 37.3237 | 0.911539 | 1.90516 | 0.00055 | 0.006645 | yes |
|  | APOBEC4 | none_lung_1d | H5N2_lung_1d | OK | 2.11999 | 4.18528 | 0.981265 | 1.1527 | 0.03485 | 0.15475 | no |
|  | APOBEC2 | none_lung_1d | H5N2_lung_1d | OK | 52.1216 | 4.79622 | -3.44191 | -2.2849 | 0.0521 | 0.202393 | no |
| H5N2 lung 3d | ADARB2 | none_lung_3d | H5N2_lung_3d | NOTEST | 0.062688 | 0 | #NAME? | 0 | 1 | 1 | no |
|  | ADARB1 | none_lung_3d | H5N2_lung_3d | OK | 7.07779 | 4.89003 | -0.533454 | -0.70947 | 0.2244 | 0.999314 | no |
|  | ADAR | none_lung_3d | H5N2_lung_3d | OK | 25.1851 | 32.554 | 0.370266 | 0.607716 | 0.29845 | 0.999314 | no |
|  | APOBEC4 | none_lung_3d | H5N2_lung_3d | OK | 1.97927 | 2.18968 | 0.145752 | 0.126537 | 0.8202 | 0.999314 | no |
|  | APOBEC2 | none_lung_3d | H5N2_lung_3d | OK | 18.7692 | 2.29969 | -3.02885 | -1.51742 | 0.25585 | 0.999314 | no |

**Table S3.** Expression profiles of ADAR and APOBEC enzymes in quail infected with H5N1 and H5N2.

|  | gene | sample_1 | sample_2 | status | value_1 | value_2 | log2(fold_change) | test_stat | p_value | q_value | significant |
| --- | --- | --- | --- | --- | --- | --- | --- | --- | --- | --- | --- |
| ileum H5N1 1d | ADARB1 | none_ileum_1d | H5N1_ileum_1d | OK | 11.8729 | 15.2672 | 0.362759 | 0.960608 | 0.09865 | 0.644287 | no |
| ileum H5N1 3d | ADARB1 | none_ileum_3d | H5N1_ileum_3d | OK | 12.9144 | 13.7036 | 0.085577 | 0.185018 | 0.7428 | 0.996923 | no |
| lung H5N1 1d | ADARB1 | none_lung_1d | H5N1_lung_1d | OK | 16.0979 | 15.3556 | -0.0681 | -0.19112 | 0.7399 | 0.998267 | no |
| lung H5N1 3d | ADARB1 | none_lung_3d | H5N1_lung_3d | OK | 16.3958 | 20.5212 | 0.323785 | 0.558083 | 0.3333 | 0.998658 | no |
| ileum H5N2 1d | ADARB1 | none_ileum_1d | H5N2_ileum_1d | OK | 11.5863 | 13.1323 | 0.180697 | 0.385763 | 0.50155 | 0.94223 | no |
| ileum H5N2 3d | ADARB1 | none_ileum_3d | H5N2_ileum_3d | OK | 13.2159 | 14.5557 | 0.139303 | 0.32435 | 0.5751 | 0.860476 | no |
| lung H5N2 1d | ADARB1 | none_lung_1d | H5N2_lung_1d | OK | 16.2861 | 19.4242 | 0.254219 | 0.61898 | 0.28165 | 0.998631 | no |
| lung H5N2 3d | ADARB1 | none_lung_3d | H5N2_lung_3d | OK | 16.6206 | 13.2592 | -0.32598 | -0.7532 | 0.19065 | 0.9987 | no |
