# Supplementary figures and images for: A comprehensive study on cellular RNA editing activity in response to infections with different subtypes of influenza a viruses
Source: BMC Genomics. 2018 Jan 19;19(Suppl 1):925. doi: 10.1186/s12864-017-4330-1 (PMC5780764; doi:10.1186/s12864-017-4330-1)

**Figure S1. Work flow of this study.**


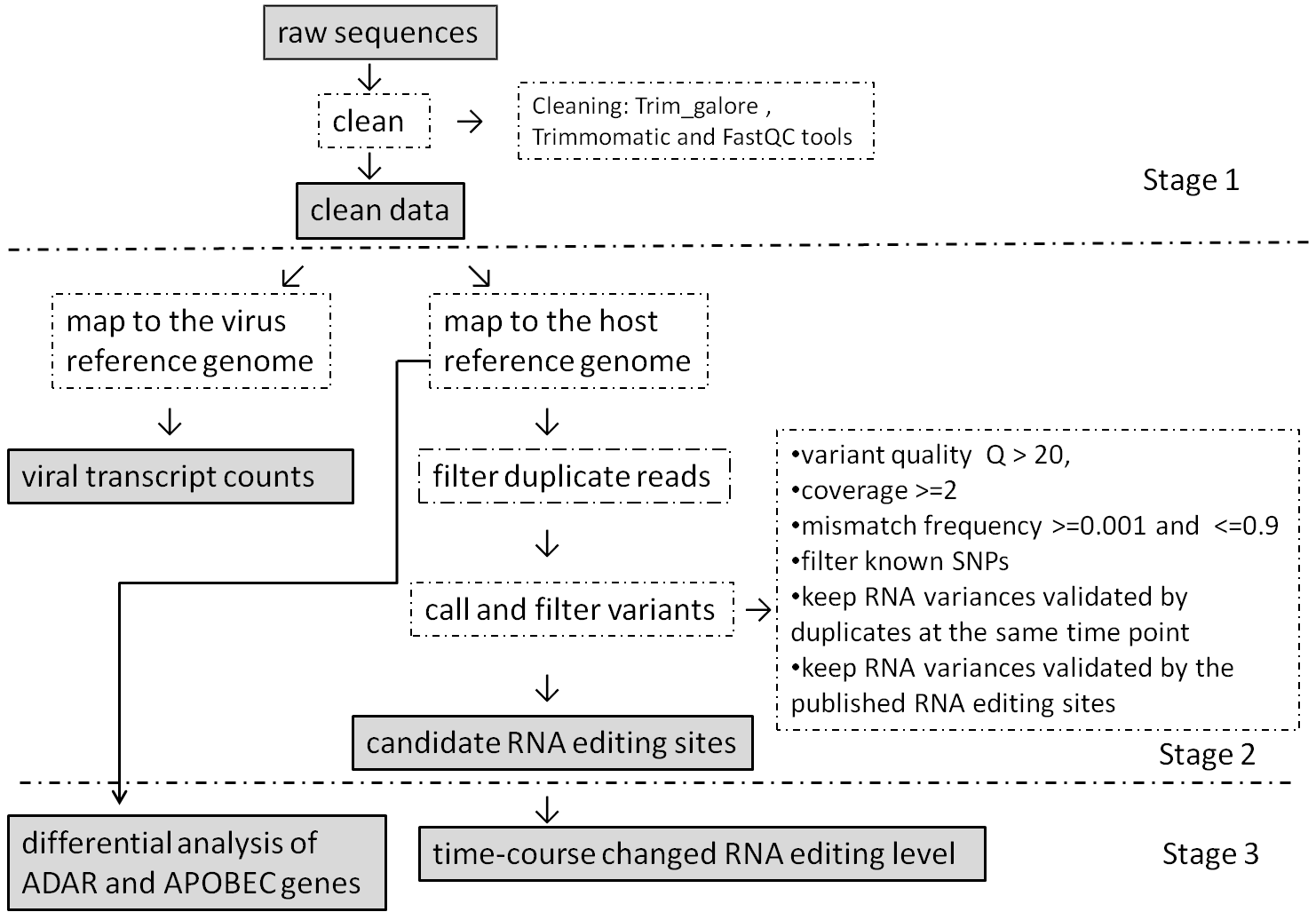

Supplement: Supplementary file 6 — Work flow of this study. (DOCX 76 kb) [file 12864_2017_4330_MOESM6_ESM.docx]
